# Lower Bone Mineral Density in Female Elite Athletes With Menstrual Dysfunction From Mixed Sports

**DOI:** 10.1155/tsm2/4969624

**Published:** 2025-01-06

**Authors:** Ida Stangerup, Anna K. Melin, Mia Lichtenstein, Lennart Friis-Hansen, Niklas R. Jørgensen, Peter Schjerling, Michael Kjaer, Kenneth H. Mertz

**Affiliations:** ^1^Department of Clinical Biochemistry, Copenhagen University Hospital, Bispebjerg-Frederiksberg, Copenhagen, Denmark; ^2^Department of Sport Science, Linnaeus University, Vaxjo/Kalmar, Sweden; ^3^Department of Psychology, University of Southern Denmark, Mental Health Services in the Region of Southern Denmark, Odense, Denmark; ^4^Department of Clinical Research, University of Southern Denmark, Odense, Denmark; ^5^Department of Clinical Biochemistry, Copenhagen University Hospital, Rigshospitalet, Copenhagen, Denmark; ^6^Department of Clinical Medicine, Faculty of Health and Medical Sciences, University of Copenhagen, Copenhagen, Denmark; ^7^Translational Research Centre, Rigshospitalet, Copenhagen, Denmark; ^8^Institute of Sports Medicine Copenhagen, Department of Orthopedic Surgery M, Copenhagen University Hospital, Bispebjerg-Frederiksberg, Copenhagen, Denmark

## Abstract

Menstrual dysfunction (MD) in female athletes might be indicative of the syndrome of relative energy deficiency in sports (REDs), associated with, e.g., impaired bone health, an increased risk of injury, and decreased performance. In the present study, we investigated differences in objective indicators of REDs, bone mineral density (BMD), and blood-based biomarkers in female elite athletes with self-reported MD or eumenorrhoea (CON) from mixed sport disciplines. Athletes reporting < 9 menstrual bleedings within the last year were recruited in the MD group, whereas eumenorrheic athletes with no symptoms of eating disorders were recruited for CON. Of the 24 athletes included, 19 completed the examinations (9 MD; 10 CON, mean age ± SD: 24.8 ± 5.5 years). Dual-energy *x-*ray absorptiometry (DXA) was used to assess body composition and BMD. Fasted rested blood sampling was performed to assess blood-based biomarkers of bone and endocrine status. Two MD athletes were excluded from the analysis due to suspected polycystic ovary syndrome. Results showed that MD was associated with lower BMD *Z*-scores across several sites compared to CON (between-group differences ± SE); whole-body *Z*-score: −1.4 ± 0.5, *p*=0.03; lumbar spine *Z*-score: −1.4 ± 0.6, *p*=0.03; proximal femur: −1.6 ± 0.6, *p*=0.02). However, no between-group differences in biomarkers of bone turnover were observed. MD was associated with lower plasma concentrations of luteinizing hormone (*p*=0.02), prolactin (*p* < 0.001), and free *T*_3_ (*p*=0.01). In conclusion, the present data indicate impairment in bone health and endocrine homeostasis in female elite athletes with current MD and underline the importance of MD as a potential indicator of REDs in female elite athletes. Furthermore, these findings call for regular screening of symptoms for early identification of athletes at risk in all sport disciplines and more education of athletes, coaches, and medical staff regarding this issue.

## 1. Background

Relative energy deficiency in sports (REDs) is a syndrome of impaired physiological and/or psychological functioning experienced by athletes caused by exposure to problematic (prolonged and/or severe) low energy availability (LEA) [1]. The detrimental outcomes include, but are not limited to, decrease in reproductive function, musculoskeletal, cardiovascular, and haematological health, which can individually and synergistically lead to impaired well-being, an increased risk of injuries, and decreased sports performance [1, 2].

The reported prevalence of LEA/REDs indicators ranges from 15% to 70% in male athletes and 23% to 80% in female athletes [2] and is frequently reported in both recreational and elite male and female athletes [3], with higher prevalence in weight-sensitive and leanness-demanding sports [2]. LEA might occur unintentionally due to factors such as misinterpretations regarding healthy and optimal sports nutrition, appetite suppression following high intensity training or disordered eating (DE) behaviour [1, 4].

Direct assessment of energy availability is time consuming and includes multiple methodological challenges such as under or over reporting of dietary intake and difficulties capturing habitual energy intake as well as measuring true exercise energy expenditure [5]. Therefore, signs and symptoms such as menstrual dysfunction (MD) may be used to screen female athletes at risk of REDs [1]. In female athletes, MD is frequently reported and may be caused by hyperandrogenism, as observed in polycystic ovary syndrome (PCOS), functional hypothalamic amenorrhoea (FHA) with persistent hypoestrogenism due to problematic LEA, or phenotypes with a combination of FHA and PCOS [6, 7].

A recent investigation among Danish elite athletes showed that 51% of female athletes not using hormonal contraceptives reported MD [8], while the frequency was 32% among Finnish athletes [9] and 60% among Nordic elite endurance athletes [4]. Given the high prevalence of MD in female athletes, research on the health risks associated with MD is of great importance.

Primary (no menarche at the age of 15 years) and secondary FHA (the absence of three or more consecutive menstrual cycles) are diagnoses of exclusion, made in the absence of identifiable organic causes. The underlying endocrine and metabolic alterations entail long-term consequences, including impaired bone health [10]. When FHA manifests at young age, it irreversibly impairs bone mass accrual since 90% of peak bone mass is achieved by the age of 18 years [11], consequently increasing risk of bone stress injuries and osteoporosis [1].

While studies have reported lower BMD in female athletes with MD compared with eumenorrheic athletes from weight-sensitive sports, e.g., endurance and aesthetic disciplines [12–16], few studies have investigated the impact of MD on BMD and blood-based biomarkers of LEA in female elite athletes from mixed sport disciplines. Blood-based biomarkers of LEA include sex hormones, markers of energy regulation (thyroid hormones), bone turnover, haematological status, and lipid status [1]. To prevent the development of serious REDs outcomes such as impaired bone health, early identification of athletes at risk of LEA is of paramount importance [17].

The aim of the present study was, therefore, to evaluate objective REDs indicators (e.g., biomarkers of bone and endocrine health) in Danish female elite athletes with self-reported MD or eumenorrhoea (CON) from mixed sport disciplines.

## 2. Methods

This study was conducted using a two-step protocol. Step one included a cross-sectional anonymous online survey regarding symptoms of LEA and DE behaviours in elite male and female athletes (*n* = 616, age 16–39 years), competing at national elite or higher competitive level within their sport discipline (Tier 4-5) [18]. The survey was distributed to elite athletes identified by the National Sports Federations and competitive clubs. Further information about the survey can be found elsewhere [19]. In total, 50 female elite athletes ≥ 18 years provided their contact information and fulfilled the inclusion criteria for step two, either self-reported MD (*n* = 23) or eumenorrhoea (*n* = 27), and were therefore invited to participate in evaluation of objective REDs indicators at the Institute of Sports Medicine at Bispebjerg and Frederiksberg Hospital, Copenhagen, Denmark. The study protocol was approved by the regional ethics committee (H-20035642), and all participants gave written consent to participate in the study. The data collection was performed between 2021 and 2022.

### 2.1. On-Line Survey

As the first step, elite athletes completed a health survey related to LEA and DE behaviour. To assess symptoms of LEA in female athletes, the LEA in Females Questionnaire (LEAF-Q) was used. Originally developed and validated in female endurance athletes (*n* = 49, 18–39 years), the LEAF-Q demonstrated a sensitivity of 78% and specificity of 90% for accurately classifying current energy availability and/or menstrual function and/or bone health [20]. Furthermore, Rogers et al. validated the LEAF-Q in a mixed-sport cohort (*n* = 75, 18–32 years) and reported an 80% sensitivity for detection of FHA and a 100% sensitivity for detection of low BMD [21]. The LEAF-Q consists of 9–25 items, depending on the respondent's answer, and assesses symptoms of LEA, injury frequency of the past year, current gastrointestinal function, hormonal contraceptive use as well as both current and past menstrual function.

To assess symptoms of DE behaviours, two online questionnaires were used: The SCOFF questionnaire [22] and the Eating Disorder Examination Questionnaire (EDE-Q) [23]. The SCOFF questionnaire is a brief five-item screening instrument developed to identify ED pathology [22], with two or more positive responses indicating an increased risk of eating disorders. A validated Danish version was used in the present study [24]. The EDE-Q has been validated in an athletic population [25]. It is a frequently used screening tool [26] and recommended to assess symptoms of DE behaviour and LEA/REDs in athletes [17]. The EDE-Q measures behavioural and cognitive symptoms of eating disorders the past 28 days and consists of 23 items divided into four subscales (restraint, eating concern, shape concern, and weight concern), and a global score averaging the subscales of ≥ 2.3 was used as cutoff for symptoms of DE behaviour [17]. SCOFF was used as a screening tool for inclusion, whereas EDE-Q was used as an outcome in the study.

### 2.2. Participants

Out of the 50 female elite athletes invited to participate in step two of the study evaluating objective indicators of REDs, 24 agreed to participate. To participate, athletes must not have used hormonal contraception for at least 3 months. Athletes presenting with oligomenorrhoea (< 9 menstrual bleedings within the last year) or secondary amenorrhoea where included for the MD group (*n* = 11). In addition, athletes with eumenorrhoea (≥ 10 menstrual bleedings the last year [27]) and low risk of DE behaviour (SCOFF score < 2) were included as controls (CON) (*n* = 13). Five athletes did not complete the examinations due to personal reasons, and two were retrospectively excluded from the analysis due to suspected PCOS (luteinizing hormone (LH): follicle-stimulating hormone (FSH) ratio > 2 and elevated free testosterone). Hence, 17 athletes (7 MD and 10 CON) were included in the analysis.

### 2.3. Evaluation of Objective REDs Indicators

Athletes arrived at Bispebjerg Hospital in an overnight fasted state between 8 and 9 a.m., having refrained from strenuous activities for the past 24 h. Eumenorrheic athletes were tested in the early follicular phase (Day 2–7 of menstruation), while MD athletes were tested when convenient.

### 2.4. Anthropometric Assessment

Height was measured without shoes to the nearest 0.1 cm using a wall-mounted stadiometer (Seca Optima, Seca, Birmingham, UK). Body mass was measured in underwear to the nearest 0.01 kg with an electronic scale (Seca 1, Model 861, Birmingham, UK). Body mass index (BMI) was calculated as weight in kilograms divided by height squared in metres (kg/m^2^). Body composition was assessed using dual-energy *x*-ray absorptiometry (DXA) (GE-Lunar Prodigy, Madison, WI, USA, EnCore software Version 16). According to best-practice protocol, the athlete's hydration status (USG) was assessed using a digital refractometer (Atago PAL-10S, Atago, Japan) prior to the DXA measurement. The same technician performed all tests with the same scanner on all participants, which was weekly calibrated using a lumbar spinal phantom. BMD was assessed through the whole-body scan, as well as at the lumbar spine (L1–L4) and femoral neck. BMD *Z*-scores were calculated based on the reference data provided in the software, and participants were categorized as having low BMD if the *Z*-score was ≤ −1.0 [1]. For the femoral neck, both sides were scanned, and the mean values were used for further analysis.

### 2.5. Blood Tests

Blood samples were drawn from the antecubital vein by a trained phlebotomist. Vacutainers not used for whole blood analysis were centrifuged at 3100 g for 5 min at 20 degrees Celsius. Leukocytes, platelets, reticulocytes, erythrocytes, haemoglobin, and haematocrit were analysed on the Sysmex XE-5000 (Sysmex Corporation, Kobe, Japan), and mean corpuscular volume (MCV) and mean corpuscular haemoglobin concentration (MCHC) were automatically calculated. Plasma ferritin, high-sensitivity c-reactive protein (hs-CRP), total calcium, 25-hydroxy-vitamin D, cortisol, prolactin, oestradiol, FSH, LH, sex hormone binding globulin (SHBG), thyroid-stimulating hormone (TSH), total and free thyroxine (T4), total and free triiodothyronine (T3), total cholesterol, high-density lipoprotein (HDL) cholesterol, and triglycerides were analysed on an automated Cobas 8000 (Roche Diagnostics, Rotkreuz, Switzerland). Low-density lipoprotein (LDL) cholesterol was calculated from total cholesterol, HDL, and triglycerides using Friedewald's formula.

Plasma osteocalcin, procollagen type 1 N propeptide (PINP), and *c*-terminal cross-linking telopeptide of type I collagen (*β*-CTX) were analysed on an automated IDS I10 (Immunodiagnostic Systems, Boldon, Tyne and Wear, UK).

Testosterone was analysed on serum using liquid chromatography-mass spectrometry (Waters UPLC-TQS LC-MSMS system, Milford, Massachusetts, USA). Free testosterone was calculated from SHBG and total testosterone.

All abovementioned biomarkers were hospital routine analyses. Hence, assays were quality assured with certified internal and external quality control material according to standard laboratory procedure.

Lastly, plasma hepcidin was analysed using the R-PLEX Human Hepcidin Antibody Set (Meso Scale Diagnostics, Rockville, Maryland, USA) since it has been suggested as a potential biomarker of LEA [28]. Samples for hepcidin were analysed in duplicates with an intraassay CV of 1.8%.

### 2.6. Statistical Analysis

Statistics were performed in GraphPad Prism Version 9.4.1 for macOS (GraphPad Software, San Diego, CA, USA). Normal distribution of continuous variables was examined visually and using the Shapiro–Wilk test. Data are presented as mean ± standard deviation (SD) or median with first and third quartiles depending on the distribution. Between-group differences were examined using unpaired *t*-tests or Mann–Whitney tests for continuous data depending on distribution. Effect sizes (ESs) were calculated using Hedge's G. A *p* value < 0.05 was considered statistically significant, and trends were discussed if *p* < 0.10.

### 2.7. Principal Component Analysis (PCA)

To qualitatively illustrate the extent to which MD and CON differed when evaluated using all available biomarkers in the dataset, and to further assess the contribution of each marker to the differences between subjects, PCA analysis was conducted. Results from all blood tests (except CRP) together with BMD, BMI, fat, and lean tissue mass were combined and scaled and centred before performing PCA using the prcomp function in *R* Version 4.3.2 [29]. Plots were generated using ggplot2 [30].

## 3. Results

### 3.1. Athlete Characteristics

Characteristics of the included athletes are summarized in Table 1. MD was associated with a higher median EDE-Q global score ([group difference ± SEM] +1.4 ± 0.5, *p*=0.03, ES = 1.56). In the MD group, four of 7 participants had EDE-Q scores ≥ 2.3, whereas no athletes in CON were over the cutoff value. Further, there was a trend towards lower BMI among MD compared to CONs (−1.5 ± 0.7 kg/m^2^, *p*=0.06, ES = −1.00). No between-group differences were observed in fat mass or lean tissue mass.

### 3.2. Bone Health

**Figure 1:**
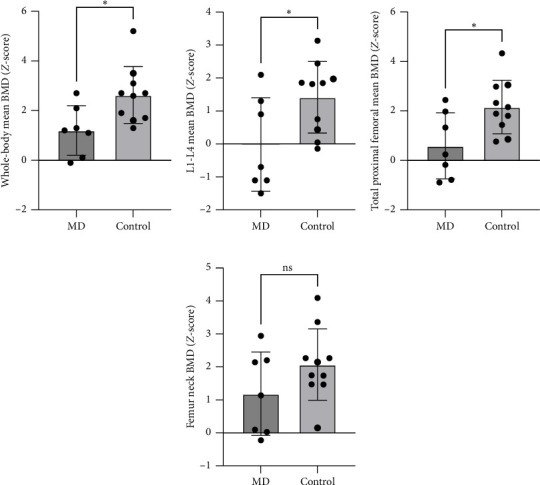
Bone mineral density in athletes with menstrual dysfunction and healthy controls. (a) Whole-body mean BMD *Z*-scores, (b) lumbar spine (L1–L4) mean BMD *Z*-score, (c) total proximal femoral mean BMD *Z*-scores, and (d) femur neck BMD *Z*-scores. Data are shown as individual data points and means ± SD. Between-group differences were tested using unpaired *t*-tests. Abbreviations: BMD, bone mineral density; MD, menstrual dysfunction.

Three of seven athletes with MD exhibited low BMD in the lumbar spine, while all CONs exhibited normal or high BMD at all measured sites. MD was associated with a significantly lower whole-body BMD *Z*-score (Figure 1(a), −1.4 ± 0.5, *p*=0.03, ES = −1.31), L1–L4 *Z*-score (Figure 1(b), −1.4 ± 0.6, *p*=0.03, ES = −1.00), and total proximal femoral *Z*-score (Figure 1(c), −1.6 ± 0.6, *p*=0.02, ES = −1.50), while femoral neck BMD Z-score did not differ between groups (Figure 1(d), −0.9 ± 0.6, *p*=0.14, ES = −0.86). No significant between-group differences were seen in plasma concentration of markers of bone formation (osteocalcin and PINP) or resorption (*β*-CTX) (Table 2).

### 3.3. Blood Tests

Athletes with MD presented with significantly lower plasma concentrations of LH and prolactin compared to CON, although all athletes for both hormones presented with concentrations within the reference range (Table 2). No significant differences in plasma concentrations of the other included sex hormones were observed between the two groups. All athletes, except for two CONs, presented with plasma oestradiol concentrations within the lowest quartile of the reference range. Among these, two MD athletes had clinically low plasmaoestradiol concentrations (0.03 and 0.05 nmol/L).

Furthermore, athletes with MD presented with lower free T3 plasma concentrations, whereas differences in concentrations of total T3, T4, and free T4 did not reach significance, despite large ESs. Five of seven athletes in the MD group and two CON athletes presented with total T3 plasma concentrations below the reference range. Individually, all athletes presented with TSH, free T3, and free T4 plasma concentrations within the reference range.

No significant between-group differences in lipid profiles or haematological parameters were otherwise observed.

### 3.4. PCA Plots

**Figure 2:**
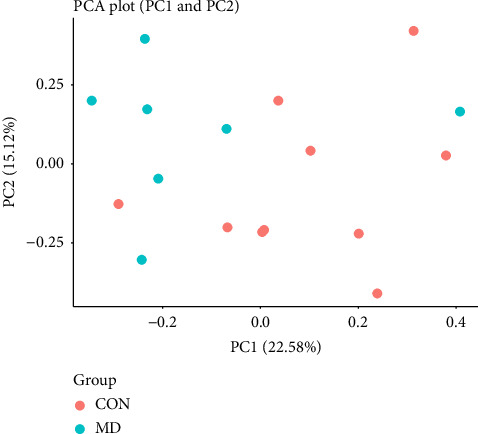
Principal component analysis plots of principal component 1 and 2. Abbreviations: CON, control group; MD, menstrual dysfunction; PC, principal component.

Examining each biomarker individually may obscure subtle effects due to confounding variations. Therefore, as part of an explorative approach, we applied the mathematical method of PCA to create principal components (“combined measures”). This model combines all real markers through a linear transformation to capture the primary combined variation in the first principal components. The first two principal components, PC1 and PC2, explained a cumulative proportion of the variation of 37.70%, meaning they captured 37.7% of the overall variation between subjects for all biomarkers. Visual inspection of the PCA plot revealed no clear separation of the two groups in PC1 and PC2. However, indications of separation between CON and MD were observed in PC1, with an MD profile showing a leftward shift on the plots, with the exception of 1 MD and 1 CON athlete (Figure 2).

**Figure 3:**
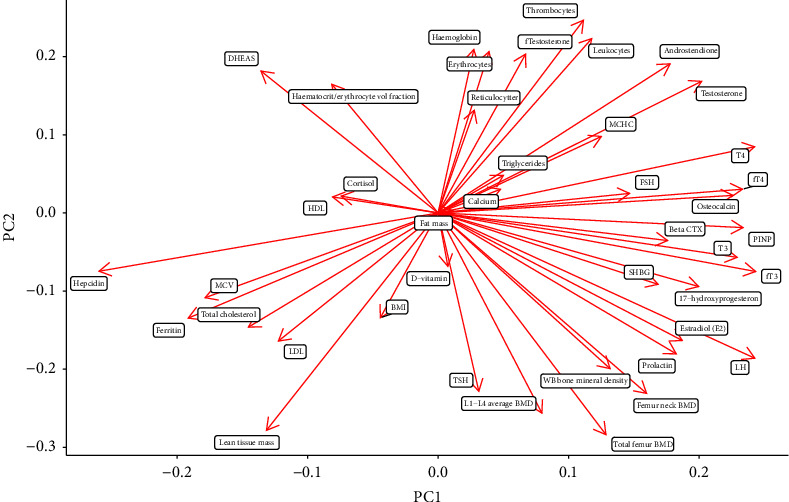
Loadings of PC1 and PC2. Abbreviations: BMD, bone mineral density; BMI, body mass index; CTX, cross-linking telopeptide of type I collagen; DHEAS, dehydroepiandrosterone sulphate; FSH, follicle-stimulating hormone; HDL, high-density lipoprotein; Hs-CRP, high-sensitivity c-reactive protein; LDL, low-density lipoprotein; LH, luteinizing hormone; MCHC, mean corpuscular haemoglobin concentration; MCV, mean corpuscular volume; MD, menstrual dysfunction; PC, principal component; PCA, principal component analysis; PINP, procollagen type 1 N propeptide; SHBG, sex hormone-binding globulin; TSH, thyroid-stimulating hormone.

Further exploring the loadings of the components (how much each marker contribute to PC1 and PC2), higher hepcidin plasma concentrations seemed to be strong indicators of an MD profile (causing leftwards shifts on the plots), whereas higher plasma concentrations of free and total T3, free and total T4, LH, and PINP seemed to be strong indicators of a CON profile, causing rightwards shift on the PCA plots (Figure 3).

## 4. Discussion

We evaluated objective REDs indicators in Danish female elite athletes with self-reported MD or eumenorrhoea (CON) from mixed sport disciplines and found that in this group of female elite athletes, MD was associated with DE behaviour and lower BMD. These findings underline the importance of regular screening to identify athletes at risk of MD and DE behaviour followed by assessment of objective indicators of REDs to prevent bone stress injury and future risk of osteoporosis [31].

### 4.1. REDs Indicators

#### 4.1.1. Bone Health

While BMD was lower in MD than in CON athletes at most measured region, the lumbar region was the only assessed region where we observed athletes with low BMD *Z*-score (≤ −1). Low lumbar *Z*-score has been associated with an increased risk of bone stress injuries at any site [32]. Participation in high-impact sports with bone-specific loading activities (e.g., soccer) increases BMD 5%–30% compared with nonathletes [33], while the lack of mechanical loading in low-impact sports (e.g., middle- and long-distance running) contributes to BMD values similar to nonathletes [34]. Three of the seven athletes with MD presented with low BMD *Z*-scores were all competing within endurance sports, whereas the four athletes with MD from more explosive sports disciplines did not exhibit low BMD *Z*-scores. Further, BMD appears to vary according to measurement site, as no significant between-group differences were observed at the femur neck. This could be due to athletes in this study generally engaging in sports disciplines involving high loading of the lower extremities, potentially masking the negative consequences of MD. This emphasizes the need for sports-specific reference data on BMD as proposed elsewhere in order not to underestimate the negative impact on bone health among athletes in high-impact and explosive sports [35].

It has previously been reported that bone metabolism is affected by both shorter and longer periods of LEA in physical active women, favouring bone resorptions over formation [36]. In this study, we included plasma osteocalcin and PINP as markers of bone formation and plasma *β*-CTX as a marker of resorption. While the between-group differences in BMD were not mirrored in significant between-group differences of included markers of bone metabolism, PCA plots indicated that osteocalcin and PINP were key contributors to a healthy CON profile.

The findings of the present study builds upon previous literature showing impaired bone health in athletes with MD, likely as a result of problematic LEA [12, 16, 37]. This underlines MD, presented as either oligo- or amenorrhoea, in athletes as a strong indicator of REDs as also stated by the newest IOC REDs consensus statement [1]. Though a strong indicator of REDs, LEA needs to be present for months before MD occurs, and years before potentially reflected in low BMD. Therefore, MD and low BMD reflect accumulated periods of problematic LEA, rather than current LEA, which again underlines the need for valid indicators of the athletes' current LEA status at the given time it is addressed. The discrepancy between the lack of difference between markers of bone turnover in the two groups and the lower bone mass in the MD group could be explained by the fact that the bone turnover markers reflect the current bone turnover at the time of blood sampling, while the BMD reflects the sum of changes in bone turnover and bone accrual for months or even years before the measurement was conducted.

#### 4.1.2. Blood Tests

The MD group exhibited significantly lower plasma LH concentrations and a tendency towards lower plasma oestradiol concentrations. These findings align with multiple studies demonstrating that LEA suppresses the hypothalamic–pituitary–gonadal axis and, if present long/severe enough, causes FHA [38, 39]. All athletes in the study presented with plasma prolactin concentrations within the normal reference range ruling out hyperprolactinemia as a cause of MD. However, significantly lower prolactin concentrations were observed in the MD group. Lower concentrations of plasma prolactin in female MD athletes compared to eumenorrheic athletes have previously been reported [40, 41]. Prolactin is a pleiotropic hormone with multiple physiological functions besides promoting lactation [42]. Release of prolactin is primarily driven by adjusting the tonic inhibiting signal from dopamine neurons. Though no classical prolactin releasing factor exists, consequently, prolactin is substantially different from the other anterior pituitary hormones, and oestradiol is still a key regulator of prolactin secretion in that it stimulates both production and release of prolactin from lactotrophs [42, 43]. Hence, the lower plasma prolactin concentrations in the MD group could be explained by the tendency towards lower plasma oestradiol concentrations.

The role of hepcidin in sports anaemia is well established [44]. However, hepcidin has additionally been proposed as a potential blood-based biomarker of LEA [28]. Although median plasma hepcidin concentration in the MD group was numerically marginally higher than in CON, and the PCA analysis suggested higher hepcidin concentration indicative of an MD profile, substantial interindividual variability in plasma concentrations for this analyte was observed. Higher concentrations of hepcidin in patients with anorexia nervosa, reflecting a chronic state of problematic LEA, compared to healthy controls have previously been reported [45]. The increase in plasma hepcidin, generated by LEA, seems to be driven through different mechanisms than the increase seen as a consequence of post exercise inflammation, involving the transcriptional coactivator peroxisome-proliferator-activated receptor gamma coactivator *α*-1 (PPARGC1 *α*), rather than interleukin-6 (IL6) [28]. Furthermore, it is important to note that an increase in the baseline plasma hepcidin concentration has recently been proposed for inclusion in the athlete's biological passport as a potential detector of autologous blood transfusion [46, 47]. Since there are multiple factors causing plasma hepcidin in elite athlete to increase, the sensitivity and specificity of hepcidin as a biomarker of LEA still needs further research.

None of the included blood-based biomarkers were able to consistently differentiate MD from CON athletes. However, five of seven MD athletes exhibited total T3 plasma concentration between 0.9 and 1.1 nmol/L, which is below the reference range (1.4–2.8 nmol/L). Therefore, total T3 seemed as the biomarker at identifying problematic LEA. Isolated low T3, referred to as “sick euthyroid”, is a well-known adaptation to both shorter and longer periods of LEA [48, 49]. The findings in this study align with the findings made by Heikura and colleagues [50] pointing out total T3 as a valid blood-based biomarker indicative of REDs. However, Heikura et al. did not measure free T3 or addressed the complete thyroid axis as done in the current study. Since plasma concentration of total T3 in most of the athletes with MD was below the reference range, whereas plasma concentration of free T3 in all athletes remained within the reference range, total T3 might be superior to the free fraction when addressing LEA and REDs. Furthermore, total T3 has the advantage of being a cheap and easy-accessible standard laboratory blood test which, at least in Denmark, is not the case with the free fraction. In the present study, blood tests and questionnaires were only performed once, providing a snapshot of the current hormonal and menstrual status. Repetitive data sampling of each athlete over a training season could provide useful information on how hormones and potential biomarkers respond to periods with fluctuating training load and energy intake, as this is the reality for many elite and subelite athletes, and how these periods correlate to oligo- and amenorrhoea as well as other symptoms of REDs.

### 4.2. Limitations

The small sample size is a limiting factor of this study. A major challenge regarding recruitment was a high prevalence of female athletes using hormonal contraceptives which served as an exclusion criterion. Hormonal contraceptive use is highly prevalent among female elite athletes [4, 8, 9]. In a recent study, Oxfeldt et al. reported that 57% of female elite athletes were using hormonal contraception, with oral contraceptives being most common [8]. The use of any hormonal contraceptive, whether progesterone alone or combined with oestrogen and regardless of the administration form, diminishes the effectiveness of addressing menstrual bleedings to screen for or identify REDs in female athletes. Due to exogenous administration of female hormones, which feeds back on the pituitary, measuring plasma hormones indicative of hypogonadotropic hypogonadism is clearly unsuitable for REDs screening in these athletes. Since hormonal contraceptives are highly prevalent in this population and mask FHA, which currently still is the strongest objective sign and symptom of REDs, future studies should consider including athletes using hormonal contraceptives to gain more knowledge on indicators of REDs in this specific population.

Finally, we did not verify that the observed MD was due to LEA. Therefore, the present findings should not be interpreted as MD being a definitive sign of REDs, as this should always be clinically verified. However, the present findings clearly highlight that MD alone is linked to several negative changes commonly associated with REDs. Therefore, MD in female athletes should be regarded as a serious medical warning sign that requires prompt attention by the athletes' coaching staff and medical team.

## 5. Conclusion and Perspectives

In conclusion, the present data show that elite athletes with self-reported MD within the last year exhibit lower BMD than athletes with regular menstrual bleedings. Furthermore, MD was observed to be associated with lower concentration of plasma LH and prolactin, as well as adaptive mechanisms in the thyroid axis reflected as isolated low total T3 plasma concentration.

Collectively, these findings underline the importance of MD as a potential sign and symptom of REDs in athletes and call for more education of athletes, coaches, and medical staff regarding this issue. Future studies should focus on identifying biomarkers of REDs in female athletes using hormonal contraceptives, as menstrual bleedings have limited usefulness in this large population of athletes. Furthermore, the present study only included female athletes, but REDs are by no means a syndrome exclusively affecting female athletes. Consequently, more research is currently needed concerning biomarkers of REDs in male athletes, and we therefore strongly encourage more studies within the field of REDs in male athletes as well.

## Figures and Tables

**Table 1:** Athlete characteristics.

	MD (*n* = 7)	Control (*n* = 10)	Between-group difference (*p* value)
Age (years)	24.2 (6.5)	26.2 (5.1)	0.48
Height (m)	1.71 (0.06)	1.70 (0.05)	0.54
Weight (kg)	60.1 (5.2)	63.0 (3.8)	0.2
BMI (kg/m2)	20.4 (1.4)	21.9 (1.5)	0.06
Body composition			
Body fat (%)	18.9 (2.7)	20.7 (3.5)	0.3
Fat mass (kg)	11.0 (1.6)	12.5 (2.3)	0.15
Lean tissue mass (kg)	47.2 (4.9)	48.2 (3.8)	0.61
Symptoms of disordered eating			
Global EDE-Q score (median (1st; 3rd quartile))	3.0 (0.4; 3.2)	0.8 (0.3; 1.2)	**0.03**
Sports distribution (N)			
Athletics (sprints)	1	1	
Athletics (middle/long distance)	1	2	
Cycling	2	0	
Kayaking	0	1	
Rugby	0	4	
Soccer	1	1	
Taekwondo	1	1	
Triathlon	1	0	

*Note:* Data are reported as (mean (SD)) unless otherwise stated. Between-group differences were tested using unpaired *t*-tests data, except for EDE-Q which was tested using Mann–Whitney test. Bold value indicates to illustrate *p* < 0.05.

Abbreviations: BMI, body mass index; EDE-Q, Eating Disorder Examination Questionnaire; MD, menstrual dysfunction.

**Table 2:** Data are shown as median values with corresponding 1^st^ and 3^rd^ quartiles.

	MD (*n* = 7)	Control (*n* = 10)	Reference range/decision limit	Effect size	Between-group difference (*p* value)
Sex hormones					
FSH (IU/L)	6.0 (4.9; 8.3)	6.6 (5.9; 8.0)	3.5–12.5^A^	−0.30	0.55
LH (IU/L)	3.6 (1.8; 4.8)	7.3 (5.0; 8.4)	0.5–18.0^A^	−1.19	**0.02**
Oestradiol (nmol/L)	0.08 (.05; 0.11)	0.15 (0.08; 0.22)	0.05–0.85^A^	−0.86	0.08
Testosterone (nmol/L)	0.75 (0.64; 0.88)	0.87 (0.72; 1.0)	0.55–1.8	−0.60	0.19
Free testosterone (nmol/L)	0.012 (0.011; 0.013)	0.014 (0.012; 0.018)	0.006–0.034	−0.67	0.15
Thyroid hormones					
TSH (mIU/L)	1.9 (1.2; 3.3)	2.8 (1.4; 3.6)	0.4–4.8	−0.16	0.75
Total T3 (nmol/L)	1.1 (1.0; 1.6)	1.4 (1.4; 1.5)	1.4–2.8	−1.11	0.09
Free T3 (pmol/L)	3.68 (3.45; 4.01)	4.30 (4.21; 4.44)	3.1–6.8	−1.42	**0.01**
Total T4 (nmol/L)	70 (67; 85)	80 (74; 87)	70–140	−0.74	0.15
Free T4 (pmol/L)	13.4 (12.4; 15.0)	14.6 (14.0; 15.8)	12.0–22.0	−0.99	0.06
Bone markers					
Osteocalcin (μg/L)	13.6 (11.2; 26.9)	17.0 (14.1; 28.7)	8.1–42.8	0.20	0.43
Beta CTX (μg/L)	846 (444; 1115)	651 (532; 865)	90–1342	0.16	0.75
PINP (g/L)	86.4 (59.0; 110.0)	84.7 (64.7; 102.5)	20–107	0.04	0.95
Total calcium (nmol/L)	2.34 (2.28; 2.39)	2.29 (2.25; 2.32)	2.15–2.51	0.90	0.09
25-Hydroxy-vitamin D (nmol/L)	70 (55; 104)	80 (74; 83.5)	> 50	−0.07	0.35
Cholesterols					
Total cholesterol (mmol/L)	4.5 (4.3; 4.7)	4.0 (3.5; 4.9)	< 5.0	0.48	0.29
HDL (mmol/L)	1.9 (1.7; 2.1)	1.9 (1.7; 2.1)	> 1.0	0.25	0.62
LDL (mmol/L)	2.3 (1.9; 2.4)	2.0 (1.2; 2.5)	< 3.0	0.37	0.28
Triglycerides (mmol/L)	0.76 (0.53; 0.88)	0.69 (0.57; 0.90)	< 2.0	0.16	0.73
Haematology					
Erythrocytes (1012/L)	4.26 (4.26; 4.78)	4.35 (4.19; 4.44)	3.94–5.16	0.41	0.49
Leukocytes (109/L)	5.3 (3.4; 6.0)	4.4 (3.9; 4.7)	3.5–8.8	0.51	0.72
Thrombocytes (109/L)	267 (198; 272)	220 (195; 249)	145–390	0.20	0.36
Reticulocytes (109/L)	55 (44; 74)	60 (51; 71)	25–99	−0.35	0.54
Haemoglobin (mmol/L)	8.1 (8.0; 8.8)	8.3 (8.0; 8.5)	7.3–9.5	0.00	1.00
Haematocrit	0.41 (0.40; 0.42)	0.40 (0.39; 0.41)	0.35–0.46	0.54	0.24
MCHC (mmol/L)	20.4 (19.6; 20.7)	20.4 (20.2; 20.9)	19.7–22.2	−0.67	0.49
MCV (fL)	94 (91; 96)	94 (91; 94)	82–98	−0.03	0.77
Others					
Hs-CRPB (mg/L)	1 (1; 1)	1 (1; 1.5)	< 10	*n/a* ^B^	0.49
Ferritin (μg/L)	48 (39; 63)	43 (28; 58)	12–300	0.19	0.49
Hepcidin (μg/L)	11 (7; 19)	7 (4; 12)	2–31	0.75	0.15
Cortisol (nmol/L)	364 (299; 460)	396 (356; 438)	133–537	0.07	0.67
Prolactin (mIU/L)	228 (159; 304)	434 (369; 474)	35–600	−2.32	**< 0.001**
SHBG (nmol/L)	47.5 (42.6; 78.3)	61.4 (37.8; 75.0)	32.4–128	0.05	0.76

*Note:* Bold values indicate to illustrate *p* < 0.05.

Abbreviations: CTX, cross-linking telopeptide of Type I collagen; FSH, follicle stimulating hormone; HDL, high-density lipoprotein; Hs-CRP, high-sensitivity c-reactive protein; LDL, low-density lipoprotein; LH, luteinizing hormone; MD, menstrual dysfunction; PINP, procollagen Type 1 N propeptide; TSH, thyroid-stimulating hormone.

^A^Follicular phase.

^B^Detection limit at 1 mg/L. Only two control athletes presented with concentrations above the detection limit, and consequently, effect size could not be calculated. Between-group differences were tested using Mann–Whitney tests.

## Data Availability

The data that support the findings of this study are available on request from the corresponding author. The data are not publicly available due to privacy or ethical restrictions.
